# Beer and Cancer

**Published:** 1902-05-17

**Authors:** 


### BEER AND CANCER.

As we mentioned a week or two ago, Mr. Jonathan Hutchinson holds that the continued use of arsenic may lead to cancer. He now goes a step further and suggests [1] that the large increase in the prevalence of cancer which has taken place during recent years may be due to the fact that modern processes of manufacture have introduced arsenic in yearly increasing quantity into various largely-used articles of diet. As an illustration of the increasing and hitherto unsuspected presence of arsenic in food he takes the case of beer. The revelations just made before the Royal Commission as to the almost inevitable presence of arsenic in all beer which is made from malt dried over coke, make it seem more than probable that for the last few centuries during which coal and coke have been gradually superseding wood for the drying of malt, the community have been habitually consuming arsenical salts. Now it is precisely during these years that cancer is supposed to have been increasing. The cases lately observed have amply illustrated the difficulty that exists in diagnosing many cases of chronic arsenical poisoning, and it is probable enough that a considerable number of cases which have in years past been registered as "peripheral neuritis," "vagabond's melasma," "pernicious anæmia," "Addison's disease," "multiple cancer of skin" and other names may in reality have had their origin in the dietetic imbibition of arsenic. "Now if, as seems proved, the continuous use of arsenic in small medicinal doses can predispose the skin to multiple cancer, there seems no reason," he says, "for doubting that it may do the same for the other tissues, and for the mucous membranes and the viscera." Of course "there must also be the constitutional tendency, the appropriate age, and in some cases the local irritation," but if it can be shown, as it apparently can, that modern methods have opened many new channels by which arsenic may obtain access to our bodies, and if the prolonged use of arsenic can so modify nutrition as to lead to the development of cancer, it seems only a legitimate use of the scientific imagination to suggest that the absorption of arsenic may be at least one cause of the modern increase in the prevalence of the disease. Mr. Hutchinson's remarks concerning the arsenical nature of the gases derived from the combustion of coal may perhaps throw some light upon the causation of "chimney-sweep's cancer." It has always been somewhat of a mystery why the irritation of soot should be so peculiarly provocative of the cancer process. But if soot contains arsenic, and arsenic causes cancer, all ought to be plain sailing.

[1] Polyclinic, May 1902.

